# Transcriptomics and eQTLs reveal inflammatory heterogeneity in the duodenal lining in coeliac disease

**DOI:** 10.1038/s41435-025-00356-0

**Published:** 2025-09-09

**Authors:** Aarón D. Ramírez-Sánchez, Stephanie Zühlke, Raúl Aguirre-Gamboa, Martijn Vochteloo, Lude Franke, Knut E. A. Lundin, Sebo Withoff, Iris H. Jonkers

**Affiliations:** 1 Department of Genetics, University Medical Center Groningen, University of Groningen, Groningen, The Netherlands; 2 K.G. Jebsen Coeliac Disease Research Centre, Institute of Clinical Medicine, University of Oslo, Oslo, Norway; 3 Department Medicine, University of Chicago, Chicago, IL USA; 4 Committee on Genetics, Genomics and Systems Biology, University of Chicago, Chicago, IL USA; 5 Oncode Institute, Utrecht, The Netherlands; 6 Department of Gastroenterology, Oslo University Hospital, Rikshospitalet, Oslo, Norway

**Keywords:** Autoimmunity, Immunogenetics, Gene expression, Quantitative trait, Mucosal immunology

## Abstract

In coeliac disease (CeD), the epithelial lining (EL) of the small intestine is severely damaged by a complex auto-inflammatory response, leading intraepithelial lymphocytes to attack epithelial cells. To understand the intestinal changes and genetic regulation in CeD, we investigated the heterogeneity in the transcriptomic profile of the duodenal EL using RNA-seq and eQTL analysis on predicted cell types. The study included duodenal biopsies from 82 patients, grouped into controls, gluten-free diet treated CeD and untreated CeD. We identified 1 862 differential expressed genes, which clustered into four sets. Two sets, one upregulated for cell cycle function (*n* = 366) and one downregulated for digestion, transmembrane transport, and laminin pathways (*n* = 543), defined three sample groups based on inflammation status: non-inflamed, mild inflammation or severe inflammation. The remaining two sets of genes were enriched for immune (*n* = 458) and extracellular matrix and barrier functions (*n* = 495) and were sufficient to classify samples into their disease conditions. Finally, deconvoluting eQTL effects from epithelial and immune cells identified 6 and 15 cell-type-mediated eQTL genes, respectively. In sum, we identified genes expressed in the duodenal EL whose expression reflect heterogeneity in CeD and that may be used as biomarkers to assess CeD condition and its mucosal and immune status.

## Introduction

Coeliac Disease (CeD) is a complex immune-mediated disorder caused by the intake of dietary gluten, a protein found in wheat, barley, and rye, in individuals with a genetic predisposition [1]. One of the hallmarks in CeD is the affected mucosa of the duodenum, which consists of two main compartments—the epithelial lining (EL) and the lamina propria—separated by the basal lamina. The duodenal mucosa is characterized by finger-like structures called villi and invaginations called crypts. The EL is a monocellular layer that covers the mucosa and contains multiple epithelial cell (EC) types. In CeD, the villus structure of the epithelium is affected and characterised by villus atrophy and crypt hyperplasia [2]. Moreover, immune cells in the form of intraepithelial lymphocytes (IELs) that normally patrol the EL for pathogens are strongly enriched in untreated CeD [3].

In CeD, IELs gain a cytotoxic phenotype resulting from a complex immune reaction. First, dietary gluten is partially digested into gliadin peptides, which are deamidated by tissue transglutaminase 2 [4, 5]. In the lamina propria, these deamidated gliadin peptides are presented by HLA-DQ2- and/or -DQ8-expressing antigen-presenting cells and recognised by gluten-specific CD4^+^ T cells, causing the latter population to expand [6, 7]. The activated gluten-specific CD4^+^ T cells promote the development of B cells and the production of auto-antibodies, and activate other T cells that respond to cytokines like IL-15 produced by ECs, move to the EL and develop into IELs [3, 8]. In CeD, IELs acquire a lymphokine killer-like activity by aberrantly expressing NK-lineage genes, including killer cell lectin-like receptor C2 (*KLRC2*, also known as *NKG2C*), natural cytotoxicity triggering receptor 1 (*NCR1*, also known as *NKp46*), and *NCR2* (also known as *NKp44*). The current thinking is that the cytotoxic IELs cause the EC damage in CeD [3, 9–12].

Once CeD patients start a gluten-free diet (GFD), some symptoms may alleviate within weeks, but overall mucosal recovery varies between patients and is only achieved in half of CeD patients after one year of GFD [13]. Crypt hyperplasia and villus atrophy gradually recover over time after gluten is excluded from the diet, and immune cells implicated in CeD pathogenesis, like gluten-specific CD4^+^ T cells in the lamina propria and cytolytic CD8^+^ IELs, decrease in numbers [14]. However, at gene expression level, biopsies from CeD patients on GFD are also distinct from controls with continuous deregulation of transport and cell cycle genes, as shown by Dotsenko et al. [15]. Understanding the causes of variation in CeD severity and recovery can therefore improve our ability to identify the underlying pathways that lead to disease (and repair) and help identify biomarkers suggestive of active disease and mucosal recovery.

To better understand the changes occurring in the EL in CeD, including after GFD, and the regulatory mechanisms that affect mucosal homeostasis in CeD, we investigated ECs and IELs in the CeD duodenal EL using RNA-seq and predicted cell-type eQTL analysis. Using gene expression profiles of EL samples, we could distinguish three inflammation states: non-inflamed, mild inflammation, and severe inflammation, and these inflammation states correlated with but were not specific to disease state. We further analysed gene expression and their interaction with SNPs associated to CeD to uncover the potential genetic contribution to disease heterogeneity.

## Materials and methods

### Ethical considerations and study design

Participants were recruited at Oslo University Hospital, including 113 participants classified as controls (CTRL, *n* = 40), treated CeD patients (TCD, *n* = 39), and untreated CeD patients (UCD, *n* = 34). CeD diagnosis followed European Society for Study of Coeliac Disease guidelines. Ethics approval was granted by the Regional Ethics Committee (6544 and 20521). Further details are given in the Supplementary Materials and Methods.

### Genotyping

DNA from blood samples and genotyped using Infinium Global Screening Array-24v1.0. Standard quality control (QC) procedures were used to remove low quality variant calls. Genotypes were imputed with the Michigan Imputation Server using the Haplotype Reference Consortium panel v1.172, as described previously [16]. Further details are given in the Supplementary Materials and Methods.

### Preparation of small intestine biopsies and FACS analysis

Biopsies were obtained by upper endoscopy and assessed for Marsh scores following standard guidelines [1]. Extra biopsies were analysed by FACS, and stored until RNA extraction. FACS data was generated using the BD LSR-II system (BD Bioscience) and analysed using FlowJo v10. Further details are given in the Supplementary Materials and Methods.

### RNA isolation and library preparation

Total RNA was extracted using the mirVana™ miRNA Isolation Kit (AM1560). The RNA was quantified and checked for integrity. Samples with a confirmed RIN > 6 and a concentration > 0.5 ng/µL were sequenced, resulting in a total of 90 samples that passed the thresholds to continue analysis. RNA library preparation was performed according to the protocol “NEBNext Ultra II Directional RNA Library Prep Kit for Illumina” (NEB #E7760S/L). NovaSeq6000 was used for clustering and DNA sequencing following manufacturer guidelines. Image analysis, base calling, and QC were performed with the Illumina data analysis pipeline RTA (version 3.4.4) and Bcl2fastq (version 2.20). Further details are given in the Supplementary Materials and Methods.

### RNA-seq quantification and QC

The adapters for sequencing were trimmed from fastQ files and aligned to build human_g1k_v37 ensembleRelease 75 reference genome using Hisat (version 0.1.5) [17] with default settings. The raw count matrix, containing 53,042 transcripts and 90 samples, was first filtered to 20,498 genes for further analysis. Based on multiple QC metrics, we removed eight outliers. The final dataset consisted of 82 samples and 20,468 genes. Further details are given in the Supplementary Materials and Methods.

### DE analysis

The DE effects of different conditions were quantified using the R package DEseq2 (version 1.34.0) [18], including sex, age, sequencing batch, total reads, RNA integrity, and %GC content as covariates in the DE model. DE effects were calculated by comparing UCD vs CTRL, TCD vs CTRL, and UCD vs TCD. DE effects were filtered on having an absolute L2FC ≥ 1 and an adjusted *p* value < 0.01. Further details are given in the Supplementary Materials and Methods.

### Clustering of DE genes and sample groups

DE genes were clustered using k-means clustering (*k* = 4) on a Euclidean distance matrix using the R package ComplexHeatmap (version 2.10.0) [19]. Samples were also clustered following a similar approach to that used for DE genes, but with *k* = 3. Further details are given in the Supplementary Materials and Methods.

### Pathway enrichment analysis

Reactome pathways [20] were used to identify the pathways or biological processes that were enriched for each set of genes. This analysis was performed using the R package clusterProfiler (version 3.14.3) [21].

### Scoring of mucosal status in samples

The mucosal status score for each sample was calculated using the methodology implemented in the R package singscore (version 1.14) and the pipeline recommended by authors [22]. As gene sets, the input included all possible combinations of DE genes included for each previously identified cluster. Using singscores to distinguish between CeD conditions, we calculated the receiver operating characteristic (ROC) curve and area under the curve (AUC) using R package pROC (version 1.18.5). Further details are given in the Supplementary Materials and Methods.

### eQTL analysis

For bulk eQTL mapping, we tested for effects between genes and CeD-associated SNPs [23, 24] located within 250 kb of a gene centre. QTL mapping was performed using an eQTL pipeline that was described previously [25]. For deconvolution of eQTL effects in cell types, we employed the method Decon-QTL [26], testing for the same effects as in the bulk eQTL mapping. As cell counts, we used proportions of major immune and epithelial cells. Cell-type-mediated QTLs were considered suggestive at a *p* value < 0.01. Further details are given in the Supplementary Materials and Methods.

### Statistical methods

Statistical analyses were performed in R (version 3.6.3) [27], unless otherwise specified. Visualisation of results was done using the R package ggplot2 (version 3.3.0) [28].

## Results

### Marsh score and disease condition are the main drivers of the transcriptomic landscape

**Fig. 1:**
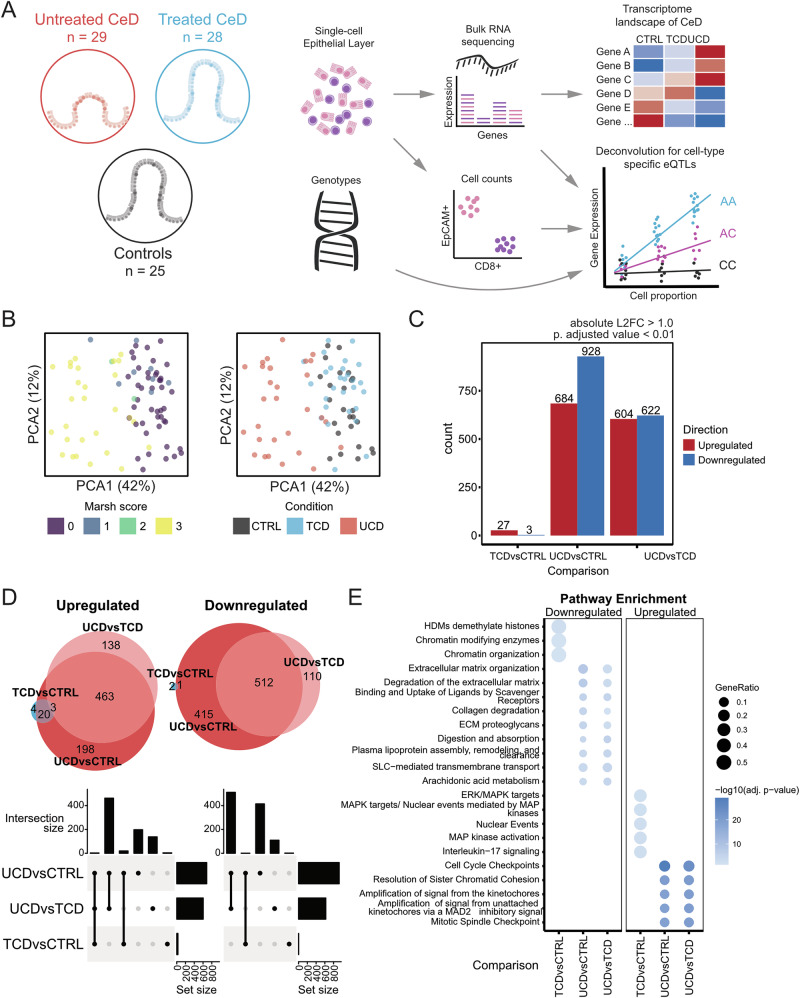
Study design and transcriptome features of duodenal epithelial lining (EL) in CeD. **A** Study design. 82 duodenal biopsies from controls (CTRL), GFD-treated CeD cases (TCD), and untreated CeD cases (UCD) were processed to isolate EL cells in single-cell suspension for cytometry (cell counts) and bulk RNA sequencing.) RNA libraries were used to study transcriptome features and derive bulk and cell-mediated eQTLs. **B** Principal component analysis (PCA) of 82 samples based on the 1 000 most variably expressed genes, coloured by Marsh score (left) and CeD condition (right). Dots represent samples; first two PCs are shown. **C** Differentially expressed (DE) genes identified in TCD vs CTRL, UCD vs CTRL, and UCD vs TCD. Genes were classified as upregulated (red) or downregulated (blue) based on Log2 Fold change (L2FC) > 1 and adjusted *p* value < 0.01. See Supplementary Table 2 for details. **D** Overlap of DE genes visualized by upregulated (left) and downregulated (right) sets using Euler diagrams (top) and Upset plots (bottom). **E** Pathway enrichment of DE genes from (**D**) using the Reactome database. Dot size represents gene ratio in pathways; shading reflects −log10(adjusted *p* value).

To study the transcriptional heterogeneity in CeD, we analysed EL isolated from intestinal biopsies from adult individuals, classified into three groups: disease controls (CTRL, patients undergoing upper endoscopy where suspicion of CeD was low but a duodenal biopsy clinically justified), treated CeD cases (TCD, CeD patients under GFD that came to planned follow-up biopsy) and untreated CeD cases (UCD, CeD patients that came to endoscopy based on serological and clinical suspicion of CeD). From these biopsy samples, we isolated cells for subsequent flow cytometry analysis, and RNA extraction to generate poly(A)-RNA-seq libraries, followed by sequencing. After quality control, we retrieved transcriptomic profiles of 25 CTRL, 28 TCD and 29 UCD samples (Table 1, extra phenotypic information from samples is provided in Supplementary table 1), in which 20,498 genes were consistently detected across all libraries (Fig. 1A).

**Table 1:** Descriptive characteristics of the cohort displayed as number (percentage) or median (interquartile range). See Supplementary table 1 for additional information.

	Total cohort	Controls	Treated CeD	Untreated CeD
Size	82	25	28	29
Marsh classification				
0	44 (54%)	25 (100%)	19 (68%)	
1	7 (9%)		7 (25%)	1 (3%)
2	2 (2%)		2 (7%)	
3	29 (35%)			28 (97%)
Sex				
Male	36 (44%)	17 (68%)	8 (29%)	11 (38%)
Female	46 (56%)	8 (32%)	20 (71%)	18 (62%)
Age (years)	44 (32–58)	43 (31–60)	45 (36–55)	41.5 (26–58)

We performed PCA to assess the variables that explain the transcriptomic landscape. We explored the first 10 principal components (PCs) by correlating them with different variables (Supplementary Figures 1–2**)**. We observed that PC1 was correlated with Marsh score, disease classification, and crypt ratio length (calculated as the ratio of expression of apolipoprotein (*APO*) A4 and marker of proliferation (*M*) KI67) [29, 30] (Fig. 1B). Sex, age, and technical characteristics (sequencing depth, sequencing batch, %GC content, and RNA integrity) were the main determinants for PCs 2–10, and these characteristics were subsequently used as covariates in all analyses (Supplementary Figure 1).

To evaluate the effects of CeD and treatment with GFD on gene expression in the EL of the small intestine, we performed differential expression analysis contrasting UCD vs CTRL, TCD vs CTRL, and UCD vs TCD. In total, we identified 1 862 differentially expressed (DE) genes (Fig. 1C, absolute Log2 Fold change (L2FC) > 1, adjusted *p* value < 0.01) (Supplementary Table 2). The UCD vs CTRL comparison showed the highest number of DE genes (*n* = 1612), followed by UCD vs TCD (*n* = 1226), whereas TCD vs CTRL exhibited only 30 DE genes. Most of the UCD vs CTRL and UCD vs TCD DE genes (%) overlap and are concordant in direction (Fig. 1D, Supplementary Figure 3). Thus, treated CeD and control individuals are similar, whereas the untreated CeD condition is associated with large changes in gene expression in cells present in the EL.

Taking direction into consideration, we performed enrichment analysis to explore the function of the DE genes (Fig. 1E, adjusted *p* value < 0.05) (Supplementary Table 3). As expected, UCD vs CTRL and UCD vs TCD exhibited remarkably similar enriched pathways. Upregulated genes such as cell-division cycle 45 (*CDC45*), minichromosome maintenance 2 (*MCM2*), and origin recognition complex 1 (*ORC1*) caused enrichment for “cell cycle pathways”. In the same comparisons, the downregulated genes collagen 4A1 (e.g., *COL4A1*), vitronectin (*VTN*), and laminin A5 (*LAMA5*) caused enrichment for “extracellular matrix function”. In addition, the “digestion and absorption pathways” were enriched via downregulation of genes encoding the digestive enzymes lactase (*LCT*) and trehalase (*TREH*), as well as solute carrier family genes (e.g., *SLC2A5*). It is likely that these observations are associated with increased proliferation of IELs and ECs in crypts and loss of differentiated absorptive ECs in the villi, which are both hallmarks of CeD [1, 31, 32].

For the TCD vs CTRL comparison, we found an enrichment for Mitogen-Activated Protein Kinase (MAPK) pathways based on the upregulated genes (e.g., *MAPK11*). This suggests that, although treated CeD samples resemble control samples, recovery may not be complete, or that TCD samples display persistent alterations in the epithelium because they previously went through an auto-inflammatory state.

### Classification of CeD states into conditions characterised by severely inflamed, mildly inflamed, or recovered epithelium

**Fig. 2:**
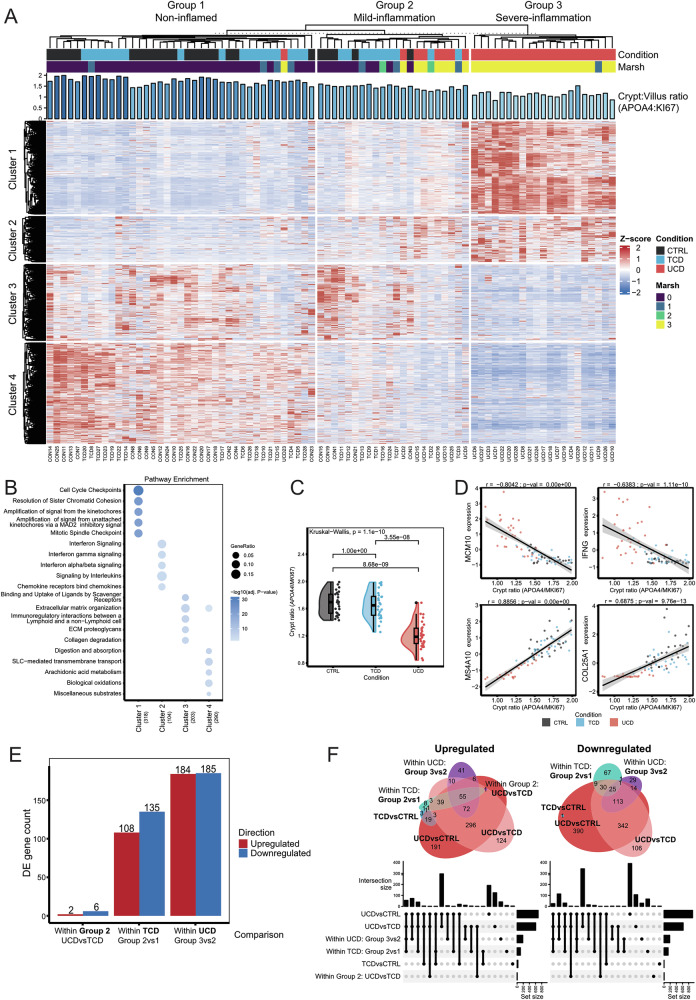
Heterogeneity of the EL gene expression allows classification of duodenal biopsies into non-inflamed, mild inflammation, and severe inflammation. **A** Heatmap of DE gene expression (*n* = 1862) across samples. Rows represent genes, columns represent samples. Genes are clustered into four clusters and three groups, shown alongside CeD condition, Marsh score, and APOA4:KI67 ratios. Scaled and centred gene expression is displayed as Z-scores. **B** Pathway enrichment analysis of gene clusters using Reactome. Top five pathways per cluster (y-axis) are shown with the number of contributing genes in brackets. **C** Statistical comparison of APOA4:KI67 ratios, a crypt length indicator, using Dunn’s test with Bonferroni correction (adjusted *p* < 0.01). **D** Correlation of representative DE genes from each cluster [1–4] with APOA4:KI67 ratios. Each plot shows VST-transformed gene expression vs. APOA4:KI67 ratio, with dot colour indicating CeD condition. (E) Overview of significant DE genes resulting from comparisons between Group 2 UCD and CTRL, within TCD (Group 2 vs 1), and within UCD (Group 3 vs 2) on the x-axis. Directions of DE are indicated by colour: upregulated (red) and downregulated (blue). See Supplementary Table 4 for complete gene lists. **F** Overlap of DE genes from all comparisons, divided into upregulated (left) and downregulated (right) sets, visualized with Euler diagrams (top) and Upset plots (bottom).

K-means clustering analysis indicated the presence of three groups in our data set using an optimal *k* = 3 determined using three different approaches (Fig. 2A, Supplementary Figure 4). For this, we used all DE genes and expected to identify groups primarily consisting of CTRLs, TCDs, and UCDs. However, when taking the distribution of samples in each group and the pathway analysis into consideration, the groups can be better defined as *non-inflamed* (group 1), *mildly inflamed* (group 2), and *severely inflamed* (group 3) than as CTRL, TCD, and UCD. This implies that inflammation status, rather than disease condition or Marsh score, is the main driver of this clustering (Fig. 2A). The non-inflamed cluster (group 1) consists of CTRLs and TCDs, as expected, whereas the mild inflammation cluster (group 2) is very heterogeneous: Some CTRL individuals are likely clustered in group 2 due to ongoing non-CeD-associated inflammation, and the TCD individuals in group 2 may not have fully recovered or do not strictly adhere to GFD. Lastly, the UCD patients in group 2 may be in an early or non-severe phase of inflammation. In the severe inflammation group (group 3), only UCD cases are observed. To further validate our inflammation groups, we tested their association with disease condition, Marsh scores, and IEL counts. We observed a strong association with both Marsh scores and disease condition (adjusted *p* value < 0.00001, Fisher-Freeman-Halton test), as well as a significant association with IEL counts (adjusted *p* value < 0.001, Fisher’s Exact Test) (Supplementary table 1). Overall, this data suggests that both the UCD and TCD groups display heterogeneous transcriptomic features and that our cohort can better be classified based on the gene expression and inflammatory state of the EL.

To identify gene functions contributing to inflammation state groups, we defined four gene clusters using K-means clustering (*k* = 4, Supplementary Figure 5). Genes in these clusters have roles in distinct biological pathways (Fig. 2B) (Supplementary Table 3):

- **Cluster 1**: Upregulated in the severely inflamed group; enriched for cell cycle and proliferation genes, including genes of the MCM protein complex, *CDC* genes, and polo-like kinase 1 (*PLK1*).
- **Cluster 2**: Upregulated in severely inflamed group and to a lesser extent in the mild inflammation group; enriched for genes associated with immune pathways and interleukin signalling, e.g., cathepsin G (*CTSG*), interleukin (*IL*) *21* *R*, *IL10*, C-X-C Motif Chemokine Ligand genes (e.g., *CXCL10* and *CXCL8*), and interferon gamma response genes including *IFNG*, signal transducer and activator of transcription 1 (*STAT1*), guanylate-binding protein genes (e.g., *GBP1*, *GBP5*, and *GBP4*), and suppressor of cytokine signalling 3 (*SOCS3*).
- **Cluster 3**: Downregulated in the severely inflamed group; enriched for genes that contribute to extracellular matrix organisation, including collagen genes (e.g., *COL1A2*, *COL3A1*, *COL18A1*, and *COL4A1*), matrix metallopeptidase genes (e.g., *MMP2* and *MMP9*), laminin (e.g., *LAMA4*), and integrin genes (e.g., *ITGA9* and *ITGA5*), and to immunoregulatory interactions, including sialic acid–binding IG-like lectin genes (e.g., *SIGLEC1*, *SIGLEC7*, and *SIGLEC9*), transmembrane immune signalling adapter *TYROBP*, natural cytotoxicity triggering receptor 2 (*NCR2*), killer cell lectin-like receptor C1 (*KLRC1*), and CD40 ligand (*CD40LG)*.
- **Cluster 4**: Downregulated in severely inflamed samples but highly expressed in non-inflamed ones. This cluster is enriched for digestion (e.g., guanylate cyclase activator 2 A (*GUCA2A*), *GUCA2B*, sucrase-isomaltase (*SI*), *LCT*, and *TREH*), SLC-mediated transmembrane transport (*SLC* genes and lipocalin 15 (*LCN15*)), and laminin interaction pathways (e.g., *LAMA1*, *LAMA5*, *LAMB2*, *LAMB3*, and *COL7A1*).

The changes in the pathways appear to be associated with specific CeD phenotypes. An increase in cell proliferation (cluster 1) of both ECs and IELs may be related to crypt hyperplasia and lymphocyte proliferation, whereas villus atrophy leads to disruption of digestion and absorption in the duodenum and could result from a problem in the basal lamina structure that contributes to a lack of mature enterocytes (cluster 4). Finally, CeD is associated with an increased type II interferon response (cluster 2), causing a disruption of extracellular matrix and immunoregulatory interactions (cluster 3).

### Differences in cell cycle, absorption, digestion, and basal lamina pathways confirm mucosal damage in UCD and TCD

Despite our finding that the overall transcriptome did not show a 1-to-1 relation with disease condition, we explored whether expression patterns of gene subsets can indicate disease status and predict the transcriptional heterogeneity observed in CTRL, UCD, and TCD. The first step is to consider the *APOA4:KI67* ratio as a proxy for villus health [29, 30, 33]. We observed that Marsh score correlated significantly with the *APOA4*:*KI67* ratio (Supplementary Figure 1). Although the *APOA4*:*KI67* ratio changes do distinguish between UCD and CTRL/TCD, we could not distinguish TCD and CTRLs (Fig. 2C, adjusted *p* value < 0.01, Dunn test, Bonferroni correction), and thus it is not useful to set a clear threshold to differentiate between the severe, mild, or non-inflamed groups. As expected, representative DE genes of each cluster also correlated well with the *APOA4*:*KI67* ratio (Fig. 2D).

Next, we performed DE analysis on genes from the groups that appeared most variable: UCD severely inflamed (group 3) vs mildly inflamed (group 2), TCD mildly inflamed (group 2) vs non-inflamed (group 1), and UCD mildly inflamed vs TCD mildly inflamed (Fig. 2E, absolute L2FC > 1, adjusted *p* value < 0.01) (Supplementary Table 4). This uncovered DE genes for each comparison, mostly between patient conditions with different inflammation levels. Additionally, to assess the putative inflammation observed within controls present in Group 2, we compared gene expression in controls classified in Group 2 versus those in Group 1. This comparison yielded 40 DEGs (Supplementary table 4), most of which were downregulated and enriched for pathways such as “GPCR ligand binding,” “Binding and Uptake of Ligands by Scavenger Receptors,” “Aquaporin-mediated transport,” “Metal ion SLC transporters,” and “Laminin interactions” (Supplementary table 5). These results suggest that some controls may exhibit signs of epithelial dysfunction, due to unknown causes. However, due to the limited number of DEGs identified and low number of controls with intermediate-inflammation, we are cautious in drawing definitive conclusions.

Most of the DE genes deregulated between UCD patients from inflammation group 3 vs 2 overlapped with DE genes of UCD vs TCD/CTRL (Fig. 2F) and had similar functions to cluster 1 and cluster 4 genes (Supplementary Fig. 6) (Supplementary Table 5): the upregulated DE gene set was enriched for cell cycle pathway genes and the downregulated gene set was enriched for ‘Digestion’ and plasma-lipoprotein-related pathways, indicating a decrease of functional enterocytes. These findings might suggest that UCD patients with mild inflammation are still in an early phase of active CeD, without full-blown damage to the EL.

To assess the heterogeneity among TCD individuals—most of whom clustered similarly to controls—and to detect DEGs associated with the CeD condition that may have been missed, we compared TCD individuals in the mild inflammation and non-inflamed groups. Most DE genes overlapped with DE genes of UCD vs TCD/CTRL and to cluster 1 and 4 (Fig. 2F). Upregulated genes were enriched for cell cycle (e.g., *CDC* genes, *MCM10*, and *CDK1*), attributable to residual crypt hyperplasia or lymphocyte expansion [31, 32, 34, 35], and downregulated genes were related to diverse transport mechanisms (e.g., *SLC* and *AQP* genes) and laminin interactions (e.g., *LAMB3*, *LAMC2*, and *LAMB2*) (Supplementary Fig. 6), which may be caused by a lack of mature enterocytes and a disruption of the basal lamina of the EL, respectively. Our results suggest that within the TCD group there are patients still in recovery. The TCD individuals in the non-inflamed group were similar to controls, indicating a healthy mucosa. However, when comparing mildly inflamed UCD vs TCD patients (group 2), we observed a small number of DE genes, which could indicate partial recovery after GFD or mild damage due to non-gluten-related inflammatory response.

Overall, the transcriptomic state of the EL is thus a good indicator of the inflammatory state and may prove helpful to further classify CeD patients as non-inflamed, mildly inflamed, or severely inflamed.

### Immune- and extracellular-matrix-associated genes classify untreated and treated CeD patients

**Fig. 3:**
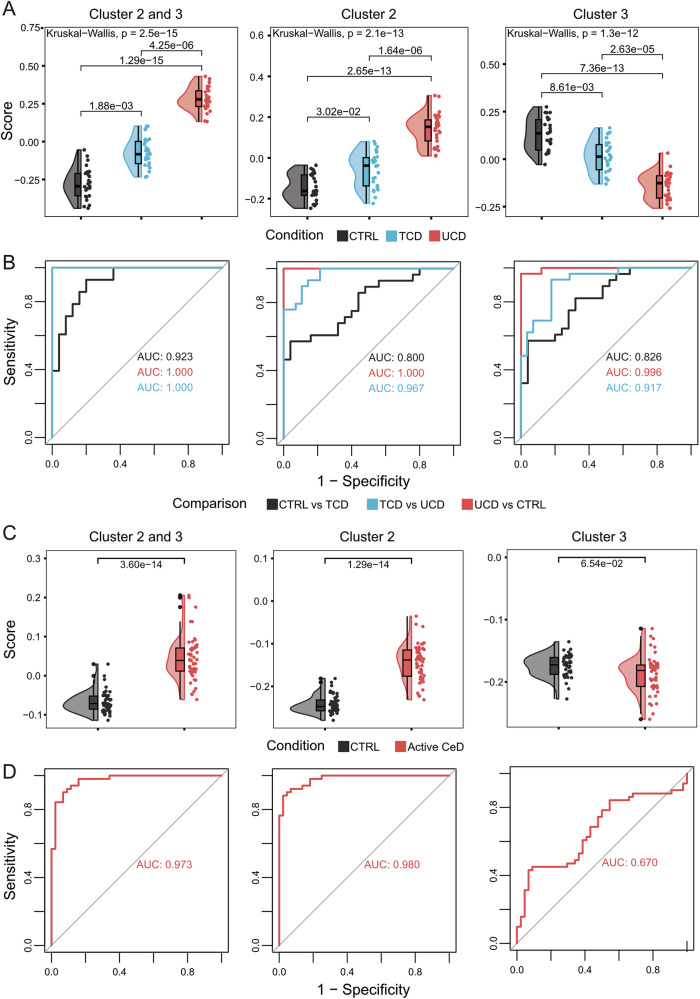
Differentially Expressed (DE) gene clusters 2 and 3 distinguish CeD status. **A** Singscores (y-axis) for clusters 2 and 3 (left), cluster 2 (centre), and cluster 3 (right), grouped by CeD condition (x-axis). Statistical analysis: Kruskal-Wallis and Dunn post-hoc tests. **B** ROC curves with AUC values for CeD condition classification using cluster-specific singscores. Comparisons: CTRL vs TCD (black), TCD vs UCD (blue), and UCD vs CTRL (red). **C** Singscores (y-axis) of an external validation cohort stratified by CeD condition (x-axis) for clusters 2 and 3 (left), cluster 2 (centre), and cluster 3 (right). Statistical tests as in panel A. **D** ROC curve and AUC for classifying external samples into CTRL or active CeD using cluster-specific singscores (red).

Genes in clusters 1 and 4 reflect inflammation and dysfunction in the small intestine but do not effectively distinguish CTRL, TCD, or UCD samples. To address this, we applied a rank-based single-sample gene set scoring method using DE gene clusters (Fig. 3A, Supplementary Table 6). This approach aims to separate samples such that active CeD cases score positively and CTRLs negatively (or vice versa) by using sets of DE genes [22]. Clusters 2 and 3 yielded the strongest distinctions, with the lowest adjusted *p* values for UCD vs CTRL (*p* = 1.29 × 10^−^¹⁵) and TCD vs CTRL (*p* = 1.88 × 10^−^³). ROC curves and AUC analysis confirmed that clusters 2 and 3 effectively differentiate the three CeD conditions (Fig. 3B) (Supplementary Fig. 8). Specifically, clusters 2 and 3 provided clear separation between UCD (median = 0.28, IQR = 0.23–0.34) and CTRL (median = −0.29, IQR = −0.36 to −0.21), with an AUC of 1, outperforming the APOA4:KI67 ratio. TCD samples scored intermediate (median = −0.08, IQR = −0.15 to 0) but retained high classification performance, with an AUC of 1 for UCD vs TCD and 0.923 for CTRL vs TCD. These results indicate that clusters 2 and 3 are robust markers for classifying CeD conditions.

To validate the ability of cluster 2 and 3 genes to define disease state, we applied them to an external transcriptomic dataset of 51 CeD patients and 44 healthy controls [36]. Combining clusters 2 and 3 achieved an AUC of 0.97, correctly predicting 43/44 CTRL and 43/51 UCD cases (Fig. 3C, D) (Supplementary Fig. 9, 10). We thus conclude that these genes in clusters 2 and 3 have the potential to distinguish between UCD, TCD, and CTRL. Moreover, since these genes are already deregulated in mild inflammation UCD individuals, with immune genes upregulated and extracellular matrix genes downregulated, we propose them as early responders in CeD inflammation.

### Deconvolution of eQTL analysis pinpoints genes affected by genetics in the immune and epithelial cell compartment

**Fig. 4:**
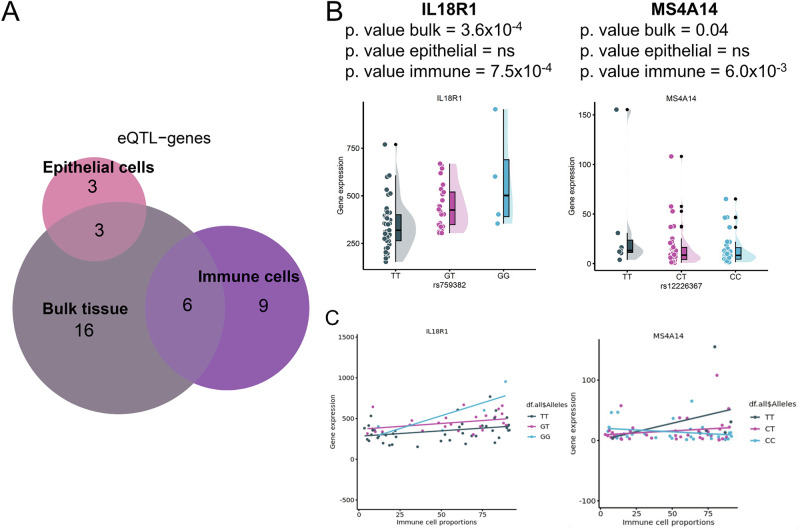
Bulk and cell-type-mediated eQTLs in CeD context. **A** Overlap of eQTL genes represented as Euler diagrams classified as bulk tissue, epithelial cell, and immune cell eQTLs. Examples of eQTLs visualised as dot plots of gene expression (y-axis) versus (**B**) genotype (x-axis) and (**C**) proportions of immune cells (x-axis). Samples coloured by genotype.

As genetics are likely to contribute to the heterogeneity between patients, we evaluated how single nucleotide polymorphisms (SNPs) associated with CeD by genome-wide association studies affect the expression of the genes in the associated genetic loci [23, 24, 37]. To minimise the multiple-testing burden, we performed *cis*-eQTL analysis using the lead SNPs in each CeD-associated locus and including genes within 250 kb of the lead SNP [24, 38]. Despite minimising the multiple-testing burden, most of the bulk and cell-type-mediated *cis*-eQTLs detected are only suggestive, likely due to power limitations given the low sample numbers. Nonetheless, we uncovered 25 eQTL genes with a suggestive significant effect (*p* value < 0.005) when analysing bulk-RNA results obtained from the EL (Fig. 4) (Supplementary Table 7). The top 5 eQTL genes were zinc finger protein 57 (*ZFP57*), two HLA genes (*HLA-G* and *HLA-K*), membrane-metalloendopeptidase-like 1 (*MMEL1*), and *IL18R1*. The first three genes are in the HLA locus on chromosome 6. The other two are located on chromosome 1 and 2, respectively. The *MMEL1* and *IL18R1* SNP-gene pairs were previously reported by the eQTLGen Consortium, which used whole-blood RNA [39], indicating that these eQTLs are not specific to duodenal tissue.

We also deconvoluted eQTL effects in both major cell types in our samples: epithelial and immune cells. For this, we applied Decon-QTL [26], which imputes cell-type-mediated eQTLs using known cell proportions (Supplementary Table 8). In total, we observed 6 EC and 15 immune cell eQTL genes, of which 3 and 9 genes, respectively, were uniquely identified in cell type specific eQTL analysis (Fig. 4A, *p* value < 0.01). *IL18R1* and *IL18RAP* were eQTL genes in both bulk tissue and immune cells, but not in ECs, indicating that these genes may be involved in CeD-associated pathways in IELs (Fig. 4B,C). Neither of these genes were DE however, indicating that genetic effects may function independently of variation in gene expression, even though both can contribute to disease state. Other immune-cell-mediated eQTL genes are membrane spanning 4-domains A14 (*MS4A14*), which is expressed in myeloid cells [40], and T cell activation RhoGTPase activating protein (*TAGAP*), a gene that we have previously found to be dysregulated in cytotoxic T cells in circulation in CeD before seroconversion [41]. The functions of the remaining eQTL genes specific to ECs or immune cells are less clear (Supplementary Table 7).

## Discussion

**Fig. 5:**
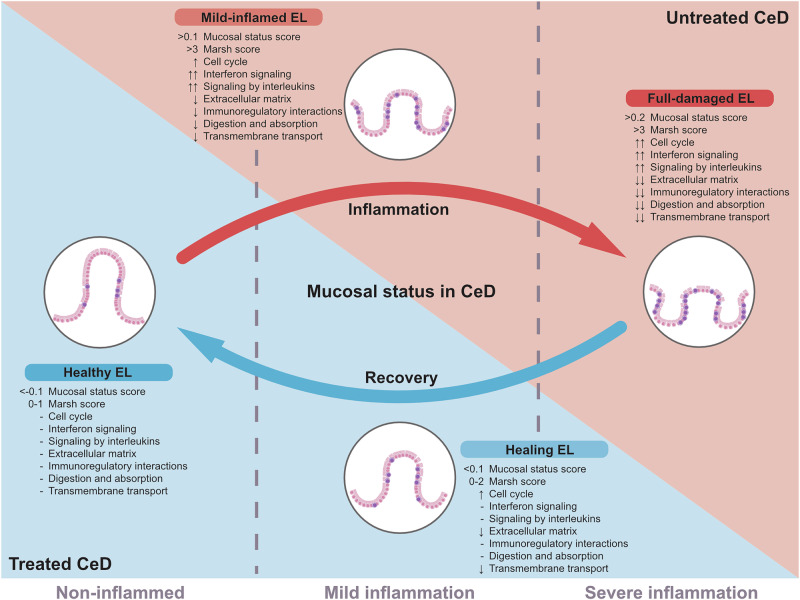
Interpretation of the transcriptomic results associated with the mucosal status of the duodenal EL in CeD. In untreated CeD (UCD), two states emerge: mild and severe inflammation. Mildly inflamed EL shows a reduced APOA4:KI67 ratio, indicating reduced villus height, with upregulated interleukin signalling, moderate cell cycle activity, and disrupted extracellular matrix and digestive functions. Severely inflamed EL exhibits extreme phenotypes: strong upregulation of pro-inflammatory pathways and the cell cycle, along with downregulation of extracellular matrix, membrane transport, metabolic, digestive, and absorptive processes. The lowest APOA4:KI67 ratios in UCD suggest severe villus atrophy. In treated CeD (TCD), EL shows signs of recovery. Mildly inflamed TCD EL resembles UCD EL with similar inflammation status but has lower Marsh scores (0–2 vs. 3 in UCD) and reduced damage and inflammation, as indicated by singscores for DE gene clusters 2 and 3. Healthy TCD EL resembles CTRL EL, with similar APOA4:KI67 ratios and Marsh scores ≤ 1, indicating a healthy phenotype. Arrows depict the direction of pathway deregulation. “-“ signifies normal expression levels.

To assess the gene expression profile of the EL in CeD specific disease states, we characterised the transcriptomic landscape of duodenal EL in treated and active CeD and control patient samples. We defined three groups based on the inflammation status (non-inflamed, mild inflammation, and severe inflammation) that were correlated with but not specific to disease state. UCD patients were found to display mild or severe inflammatory transcriptional features, whereas TCD patients exhibited a transcriptional phenotype similar to that of CTRL individuals or to UCD patients with mild inflammation. Two clusters of genes enriched for immune and extracellular matrix and barrier function (Fig. 5) yielded the best classification into specific conditions (CTRL, TCD, and UCD). Overall, we observed a marked heterogeneity in gene expression profiles of the EL of UCD and TCD individuals.

Determination of DE genes from duodenal EL can help uncover the genes and pathways that are involved in the tissue damage associated with CeD and shed light on the causes of the phenotypical heterogeneity in the disease. Our data uncovered genes explaining inflammation status (and presumably villous atrophy) and disease severity. UCD cases are characterised by upregulation of cell cycle and proliferation genes (e.g., *MCM* and *CDC* genes, *PLK1*, and *CLK1*) and downregulation of digestive (e.g., *GUCA2A*, *GUCA2B*, *SI*, *LCT*, and *TREH*), transmembrane transport (*SLC* genes), and basal lamina genes (e.g., *LAMA1*, *LAMA5*, *LAMB2*, *LAMB3*, and *COL7A1*) (Fig. 2). DE genes were further classified into four clusters, leading us to distinguish cluster 1 as enriched for cell proliferation functions and cluster 4 as enriched for digestion, absorption, and laminin functions. These pathways are mainly deregulated in UCD and are indicative of an expanding IEL population and EC proliferation that in turn may result in crypt hyperplasia [31, 32, 35, 42]. The downregulation of digestive, transport, and laminin interaction pathways suggest a loss of intestinal epithelial functions, perhaps due to the loss of enterocytes due to villous atrophy. Downregulation of transport-related genes is in line with previous observations by Laforenza et al. and Dotsenko et al, who found alteration of expression of aquaporin genes (*AQP7, AQP10*, and *AQP11*) and solute carrier genes (*SLC5A1* and *SLC15A1*) in the same direction [15, 43]. Similarly, in intestinal biopsies of CeD patients, Veberke et al. found a weaker staining of proteins related to extracellular matrix, such as collagen IV, laminin and fibronectin, indicating a concordance with the trends we observe in our study [44]. In our TCD cases, we observed that increased expression of cell cycle and proliferation genes, along with decreased expression of digestive, transmembrane transport, and basal lamina genes, differentiates the non-inflamed from the mildly inflamed states. This expression pattern suggests that the epithelial layer and enterocyte function remain incompletely restored. Dotsenko et al. observed similar pathway alterations when they compared long-term GFD-treated CeD patients to healthy controls, concluding that the treated patients still lacked a fully healthy intestine [15]. Overall, cluster 1 and cluster 4 genes recapitulate the mucosal inflammation status of EL in CeD, making it possible to identify mild or severe inflammation in untreated CeD cases and non-inflamed or mild inflammation in treated CeD cases.

Leonard et al. [45] also studied the duodenal transcriptome of UCD, TCD patients, and CTRLs, observing that most DE genes (89%) corresponded to the comparisons of active CeD biopsies versus any other condition. They identified DE genes and enriched pathways similar to our findings, including genes associated with CeD (*IFNG, CDC45*, and *MCM*2), interleukins (*IL10* and *IL17A*), chemokines (*CXCL3, CXCL9, CXCL10*, and *CXCL11*), and cell adhesion molecules (*CLDN18*). However, when comparing TCD cases versus controls, they reported more DE genes (290 vs 30 DE genes). Similarly, Dotsenko et al. found 167 DE genes in the same comparison [15]. We speculate that this divergence may be attributed to the sample size, processing/preparation methods, or tissue cell composition. For instance, this study focuses on the epithelial lining (EL), while Leonard et al. [45] analyzed whole duodenal biopsies, which include the lamina propria. The lamina propria may better recapitulate the residual immune dysregulation after GFD in CeD cases. Indeed, immune-associated genes were not enriched in our DE analysis within TCD (mild inflammation vs non-inflamed) and within UCD (severe vs mild inflammation), supporting the idea that lamina propria may be a better context to observe the immune response before and after GFD [46].

DE genes in clusters 2 and 3 were consistently upregulated and downregulated, respectively, in UCD, regardless of disease severity, and effectively distinguished UCD from CTRL and TCD (Fig. 3). These genes may play a role in CeD onset, being deregulated independently of intestinal dysfunction and proliferation. Cluster 2 genes are enriched in immune-related pathways, including interferon gamma (e.g., *IFNG*, *STAT1*, and *GBP1*) and interleukin signalling (e.g., *IL21R*, *IL17A*, and *IL10*). They also include highly upregulated genes in CeD-associated IELs (e.g., *KLRC2, RTKN2, BUB1B, TNFRSF9*, and *CISH)* [11, 12, 47] and genes like *IFNG*, *GBP5*, *CXCL10*, and *UBD*, known to be upregulated in active CeD prior to diagnosis [48]. Cluster 3 genes are enriched in extracellular matrix organization (e.g., *COL23A1*, *MMP2*, and *DCN*) and immunoregulatory interactions (e.g., *TYROBP*, *SIGLEC1*, and *CD40LG*). Some downregulated cluster 3 genes (e.g., *GNLY*, *KLRC1*, *TYROBP, FCER1G, GZMK*, and *SH2D1B*) are implicated in natural IEL function in healthy mucosa [12]. Cluster 2 and 3 genes clearly distinguish UCD from TCD and CTRLs, while cluster 1 and 4 are more indicative of villous atrophy and tissue damage in UCD or incomplete recovery in TCD (Supplementary Fig. 7). Finally, TCD singscores for clusters 2 and 3 fall between CTRL and UCD, suggesting incomplete mucosal recovery, consistent with reports that CeD-associated IELs persist in the EL after starting a GFD [12, 47]. Thus, cluster 2 and 3 represent a novel means to capture mucosal status, immune processes, and IEL populations, offering a valuable tool for understanding CeD development. Furthermore, our results demonstrate that transcriptomic profiling of biopsies reveals heterogeneity among treated patients that is not apparent through histopathological assessment alone. Based on this, we propose these genes can be used to develop a scoring system that aids in disease diagnosis. This approach may also help identify patients who, despite adhering to a GFD, still exhibit signs of inflammation. This finding is particularly relevant for cases with an unclear diagnosis.

Our eQTL and deconvolution analysis resulted in more immune-cell-mediated hits than EC-mediated hits. *IL18R1*:rs759382 is both a bulk eQTL and an immune-mediated eQTL, and it was reported previously as a blood eQTL [39]. Although *IL18R1* is not DE, its ligand, *IL18*, was downregulated in UCD and present in cluster 4. IL18R is found on the surface of several cell types, including ECs, dendritic cells, and subsets of lymphocytes, and thus its interaction with IL18 may be altered because of genetic associations in the context of CeD. IL18 and IL18R have pleiotropic functions in maintaining inflammation in CeD [49, 50] and affect epithelial barrier function in colitis [51]. The eQTL *MS4A14*:rs12226367 was only found in the immune-compartment-mediated eQTL analysis. *MS4A14* is expressed mainly in monocytes and myeloid cells and is located in the same locus as other members of the MS4A family [40]. Although to our knowledge this is a novel eQTL effect, rs12226367 was previously associated to the expression of *MS4A6A* [52]. Overall, we obtained a relatively small number of eQTL genes, likely due to the lack of power because of the limited size of our cohort. Moreover, to standardise the eQTL analysis and eliminate the influence of CeD status, we controlled for CeD condition without considering inflammation status. This approach may have hampered the analysis of genetic effects on gene expression in our data. Conducting additional eQTL analyses in larger and more precisely stratified cohorts for CeD, and incorporating factors such as inflammation status, could potentially address this limitation. This would enable a more thorough exploration of the interactions between cell-type-mediated eQTL effects and CeD condition.

In conclusion, using cell cycle, digestive, absorptive, and basal lamina genes, we can stratify CeD patients based on their inflammation/villus damage status. Remarkably, despite heterogeneity in UCD and TCD cases, we were able to accurately classify untreated CeD patients, treated CeD patients, and controls based on immune and extracellular matrix genes, which we suggest play an important role in CeD pathophysiology. Overall, we identified genes expressed in the duodenal EL whose expression might be used as biomarkers to assess CeD condition and its mucosal and immune status.

## Supplementary information

Supplementary Materials and Methods

Overview Supplemental Data

Supplementary Figures

Supplementary Data

Supplementary Table 1

Supplementary Table 2

Supplementary Table 3

Supplementary Table 4

Supplementary Table 5

Supplementary Table 6

Supplementary Table 7

Supplementary Table 8

## Data Availability

All code and scripts used to generate the results and figures are available on Github (https://github.com/umcg-immunogenetics/CeD_EpithelialLining_RNAseq_Ramirez-Sanchez_2024).

## Data availability

The RNA-seq count tables and FACS counts required to reproduce this study are provided as supplementary files. The raw RNA-seq and genotypes supporting this study will be available from the European Genome-Phenome Archive under accession number (in submission).

## References

[1] Al-Toma A, Volta U, Auricchio R, Castillejo G, Sanders DS, Cellier C, et al. European Society for the Study of Coeliac Disease (ESsCD) guideline for coeliac disease and other gluten-related disorders. United Eur Gastroenterol J. 2019;7:583–613. 10.1177/2050640619844125 PMC6545713 31210940

[2] Iversen R, Sollid LM. The immunobiology and pathogenesis of celiac disease. Annu Rev Pathol Mech Dis. 2023;18:47–70. 10.1146/annurev-pathmechdis-031521-032634 36067801

[3] Mayassi T, Jabri B. Human intraepithelial lymphocytes. Mucosal Immunol. 2018;11:1–9. 29674648 10.1038/s41385-018-0016-5 PMC6178824

[4] Arentz-Hansen H, Körner R, Molberg Ø, Quarsten H, Vader W, Kooy YMC, et al. The intestinal T cell response to α-gliadin in adult celiac disease is focused on a single deamidated glutamine targeted by tissue transglutaminase. J Exp Med. 2000;191:603–12. 10684852 10.1084/jem.191.4.603 PMC2195837

[5] Simon-Vecsei Z, Király R, Bagossi P, Tóth B, Dahlbom I, Caja S, et al. A single conformational transglutaminase 2 epitope contributed by three domains is critical for celiac antibody binding and effects. Proc Natl Acad Sci USA. 2012;109:431–6. 22198767 10.1073/pnas.1107811108 PMC3258628

[6] Molberg, Kett K, Scott H, Thorsby E, Sollid LM, Lundin KEA. Gliadin specific, HLA DQ2-restricted T cells are commonly found in small intestinal biopsies from coeliac disease patients, but not from controls. Scand J Immunol. 1997;46:103–8. 9246215

[7] Lundin KE, Scott H, Hansen T, Paulsen G, Halstensen TS, Fausa O, et al. Gliadin-specific, HLA-DQ(alpha 1*0501, beta 1*0201) restricted T cells isolated from the small intestinal mucosa of celiac disease patients. J Exp Med. 1993;178:187–96. 8315377 10.1084/jem.178.1.187 PMC2191064

[8] Mesin L, Sollid LM, Di Niro R. The intestinal B-cell response in celiac disease. Front Immunol. 2012;3:1–12. 23060888 10.3389/fimmu.2012.00313 PMC3463893

[9] Cerf-Bensussan N, Guy-Grand D, Griscelli C. Intraepithelial lymphocytes of human gut: isolation, characterisation and study of natural killer activity. Gut. 1985;26:81–8. 3871194 10.1136/gut.26.1.81 PMC1432399

[10] Jabri B, De Serre NPM, Cellier C, Evans K, Gache C, Carvalho C, et al. Selective expansion of intraepithelial lymphocytes expressing the HLA-E- specific natural killer receptor CD94 in celiac disease. Gastroenterology. 2000;118:867–79. 10784586 10.1016/S0016-5085(00)70173-9 PMC7095198

[11] Kornberg A, Botella T, Moon CS, Rao S, Gelbs J, Cheng L, et al. Gluten induces rapid reprogramming of natural memory αβ and γδ intraepithelial T cells to induce cytotoxicity in celiac disease. Sci Immunol. 2023;8:1–29. 10.1126/sciimmunol.adf4312 PMC10481382 37450575

[12] Atlasy N, Bujko A, Bækkevold ES, Brazda P, Janssen-Megens E, Lundin KEA, et al. Single cell transcriptomic analysis of the immune cell compartment in the human small intestine and in celiac disease. Nat Commun. 2022;13:4920. 35995787 10.1038/s41467-022-32691-5 PMC9395525

[13] Hære P, Høie O, Schulz T, Schönhardt I, Raki M, Lundin KEA. Long-term mucosal recovery and healing in celiac disease is the rule—not the exception. Scand J Gastroenterol. 2016;51:1439–46. 27534885 10.1080/00365521.2016.1218540

[14] Leonard MM, Silvester JA, Leffler D, Fasano A, Kelly CP, Lewis SK, et al. Evaluating responses to gluten challenge: a randomized, double-blind, 2-dose gluten challenge trial. Gastroenterology. 2021;160:720–733.e8. 33130104 10.1053/j.gastro.2020.10.040 PMC7878429

[15] Dotsenko V, Oittinen M, Taavela J, Popp A, Peräaho M, Staff S, et al. Genome-wide transcriptomic analysis of intestinal mucosa in celiac disease patients on a gluten-free diet and postgluten challenge. Cell Mol Gastroenterol Hepatol. 2021;11:13–32. 32745639 10.1016/j.jcmgh.2020.07.010 PMC7593586

[16] Das S, Forer L, Schönherr S, Sidore C, Locke AE, Kwong A, et al. Next-generation genotype imputation service and methods. Nat Genet. 2016;48:1284–7. 27571263 10.1038/ng.3656 PMC5157836

[17] Kim D, Langmead B, Salzberg SL. HISAT: a fast spliced aligner with low memory requirements. Nat Methods. 2015;12:357–60. 25751142 10.1038/nmeth.3317 PMC4655817

[18] Love MI, Huber W, Anders S. Moderated estimation of fold change and dispersion for RNA-seq data with DESeq2. Genome Biol. 2014;15:550 25516281 10.1186/s13059-014-0550-8 PMC4302049

[19] Gu Z. Complex heatmap visualization. iMeta. 2022;1:e43 38868715 10.1002/imt2.43 PMC10989952

[20] Fabregat A, Jupe S, Matthews L, Sidiropoulos K, Gillespie M, Garapati P, et al. The reactome pathway knowledgebase. Nucleic Acids Res. 2018;46:D649–55. 29145629 10.1093/nar/gkx1132 PMC5753187

[21] Yu G, Wang LG, Han Y, He QY. ClusterProfiler: an R package for comparing biological themes among gene clusters. OMICS J Integr Biol. 2012;16:284–7. 10.1089/omi.2011.0118 PMC3339379 22455463

[22] Foroutan M, Bhuva DD, Lyu R, Horan K, Cursons J, Davis MJ. Single sample scoring of molecular phenotypes. BMC Bioinforma. 2018;19:404. 10.1186/s12859-018-2435-4 PMC6219008 30400809

[23] Ricaño-Ponce I, Zhernakova DV, Deelen P, Luo O, Li X, Isaacs A, et al. Refined mapping of autoimmune disease associated genetic variants with gene expression suggests an important role for non-coding RNAs. J Autoimmun. 2016;68:62–74. 26898941 10.1016/j.jaut.2016.01.002 PMC5391837

[24] Ricaño-Ponce I, Gutierrez-Achury J, Costa AF, Deelen P, Kurilshikov A, Zorro MM, et al. Immunochip meta-analysis in European and Argentinian populations identifies two novel genetic loci associated with celiac disease. Eur J Hum Genet. 2020;28:313–23. 31591516 10.1038/s41431-019-0520-4 PMC7028987

[25] Zhernakova DV, Deelen P, Vermaat M, van Iterson M, van Galen M, Arindrarto W, et al. Identification of context-dependent expression quantitative trait loci in whole blood. Nat Genet. 2017;49:139–45. 27918533 10.1038/ng.3737

[26] Aguirre-Gamboa R, de Klein N, di Tommaso J, Claringbould A, Võsa U, Zorro M, et al. Deconvolution of bulk blood eQTL effects into immune cell subpopulations. bioRxiv. 2019;5:1–23. 10.1186/s12859-020-03576-5 PMC7291428 32532224

[27] R Core Team. R: A Language and Environment for Statistical Computing. R Foundation for Statistical Computing, Vienna, Austria; 2019.

[28] Wickham H. ggplot2: elegant graphics for data analysis. Springer-Verlag, New York, USA; 2016.

[29] Taavela J, Viiri K, Popp A, Oittinen M, Dotsenko V, Peräaho M, et al. Histological, immunohistochemical and mRNA gene expression responses in coeliac disease patients challenged with gluten using PAXgene fixed paraffin-embedded duodenal biopsies. BMC Gastroenterol. 2019;19:189. 31730447 10.1186/s12876-019-1089-7 PMC6858741

[30] Taavela J, Viiri K, Välimäki A, Sarin J, Salonoja K, Mäki M, et al. Apolipoprotein A4 defines the villus-crypt border in duodenal specimens for celiac disease morphometry. Front Immunol. 2021;12:1–10. 10.3389/fimmu.2021.713854 PMC8358775 34394117

[31] Schumann M, Siegmund B, Schulzke JD, Fromm M. Celiac disease: role of the epithelial barrier. CMGH Cell Mol Gastroenterol Hepatol. 2017;3:150–62. 28275682 10.1016/j.jcmgh.2016.12.006 PMC5331784

[32] Halstensen TS, Brandtzaeg P. Activated T lymphocytes in the celiac lesion: Non-proliferative activation (CD25) of CD4 + α/β cells in the lamina propria but proliferation (Ki-67) of α/β and γ/δ cells in the epithelium. Eur J Immunol. 1993;23:505–10. 8094672 10.1002/eji.1830230231

[33] Kurppa K, Taavela J, Saavalainen P, Kaukinen K, Lindfors K. Novel diagnostic techniques for celiac disease. Expert Rev Gastroenterol Hepatol. 2016;10:795–805. 26838683 10.1586/17474124.2016.1148599

[34] Dewar DH, Ciclitira PJ. Clinical features and diagnosis of celiac disease. Gastroenterology. 2005;128:19–24. 10.1053/j.gastro.2005.02.010 15825122

[35] Gracz AD, Fuller MK, Wang F, Li L, Stelzner M, Dunn JCY, et al. Brief Report: CD24 and CD44 mark human intestinal epithelial cell populations with characteristics of active and facultative stem cells. Stem Cells. 2013;31:2024–30. 23553902 10.1002/stem.1391 PMC3783577

[36] Abadie V, Kim SM, Lejeune T, Palanski BA, Ernest JD, Tastet O, et al. IL-15, gluten and HLA-DQ8 drive tissue destruction in coeliac disease. Nature. 2020;578:600–4. 32051586 10.1038/s41586-020-2003-8 PMC7047598

[37] Trynka G, Hunt KA, Bockett NA, Romanos J, Mistry V, Szperl A, et al. Dense genotyping identifies and localizes multiple common and rare variant association signals in celiac disease. Nat Genet. 2011;43:1193–201. 22057235 10.1038/ng.998 PMC3242065

[38] van der Graaf A, Zorro MM, Claringbould A, Võsa U, Aguirre-Gamboa R, Li C, et al. Systematic prioritization of candidate genes in disease loci identifies TRAFD1 as a master regulator of IFNγ signaling in celiac disease. Front Genet. 2021;11:1–16. 10.3389/fgene.2020.562434 PMC7868554 33569077

[39] Võsa U, Claringbould A, Westra HJ, Bonder MJ, Deelen P, Zeng B, et al. Large-scale cis- and trans-eQTL analyses identify thousands of genetic loci and polygenic scores that regulate blood gene expression. Nat Genet. 2021;53:1300–10. 34475573 10.1038/s41588-021-00913-z PMC8432599

[40] Silva-Gomes R, Mapelli SN, Boutet Mastrid, Mattiola I, Sironi M, Grizzi F, et al. Differential expression and regulation of MS4A family members in myeloid cells in physiological and pathological conditions. J Leukoc Biol. 2022;111:817–36. 34346525 10.1002/JLB.2A0421-200R PMC9290968

[41] Ramírez-Sánchez AD, Chu X, Modderman R, Kooy-Winkelaar Y, Koletzko S, Korponay-Szabó IR, et al. Single-cell RNA sequencing of peripheral blood mononuclear cells from pediatric coeliac disease patients suggests potential pre-seroconversion markers. Front Immunol. 2022;13:843086. 35371081 10.3389/fimmu.2022.843086 PMC8964997

[42] Veress B, Franzén L, Bodin L, Borch K. Duodenal intraepithelial lymphocyte-count revisited. Scand J Gastroenterol. 2004;39:138–44. 15000275 10.1080/00365520310007675

[43] Laforenza U, Miceli E, Gastaldi G, Scaffino MF, Ventura U, Fontana JM, et al. Solute transporters and aquaporins are impaired in celiac disease. Biol Cell. 2010;102:457–67. 20415666 10.1042/BC20100023

[44] Verbeke S, Gotteland M, Fernandez M, Bremer J, Rios G, Brunser O. Basement membrane and connective tissue proteins in intestinal mucosa of patients with coeliac disease. J Clin Pathol. 2002;55:440–5. 12037027 10.1136/jcp.55.6.440 PMC1769663

[45] Leonard MM, Bai Y, Serena G, Nickerson KP, Camhi S, Sturgeon C, et al. RNA sequencing of intestinal mucosa reveals novel pathways functionally linked to celiac disease pathogenesis. PLoS ONE. 2019;14:e0215132. 10.1371/journal.pone.0215132 PMC6472737 30998704

[46] Yu X, Vargas J, Green PHR, Bhagat G. Innate lymphoid cells and celiac disease: current perspective. Cmgh. 2021;11:803–14. 33309944 10.1016/j.jcmgh.2020.12.002 PMC7851184

[47] Mayassi T, Ladell K, Gudjonson H, McLaren JE, Shaw DG, Tran MT, et al. Chronic inflammation permanently reshapes tissue-resident immunity in celiac disease. Cell. 2019;176:967–981.e19. 30739797 10.1016/j.cell.2018.12.039 PMC6667191

[48] Bragde H, Jansson U, Fredrikson M, Grodzinsky E, Söderman J. Celiac disease biomarkers identified by transcriptome analysis of small intestinal biopsies. Cell Mol Life Sci. 2018;75:4385–401. 30097691 10.1007/s00018-018-2898-5 PMC6208765

[49] Yasuda K, Nakanishi K, Tsutsui H. Interleukin-18 in health and disease. Int J Mol Sci. 2019;20:649. 30717382 10.3390/ijms20030649 PMC6387150

[50] León AJ, Garrote JA, Blanco-Quirós A, Calvo C, Fernández-Salazar L, Del Villar A, et al. Interleukin 18 maintains a long-standing inflammation in coeliac disease patients. Clin Exp Immunol. 2006;146:479–85. 17100768 10.1111/j.1365-2249.2006.03239.x PMC1810422

[51] Nowarski R, Jackson R, Gagliani N, de Zoete MR, Palm NW, Bailis W, et al. Epithelial IL-18 equilibrium controls barrier function in colitis. Cell. 2015;163:1444–56. 26638073 10.1016/j.cell.2015.10.072 PMC4943028

[52] Jansen R, Hottenga JJ, Nivard MG, Abdellaoui A, Laport B, de Geus EJ, et al. Conditional eQTL analysis reveals allelic heterogeneity of gene expression. Hum Mol Genet. 2017;26:1444–51. 28165122 10.1093/hmg/ddx043 PMC6075455

