# Supplementary material for: Transcriptomics and eQTLs reveal inflammatory heterogeneity in the duodenal lining in coeliac disease
Source: Genes Immun. 2025 Sep 9;26(5):519–30. doi: 10.1038/s41435-025-00356-0 (PMC12527925; doi:10.1038/s41435-025-00356-0)
Supplement: Supplementary file 1 — Supplementary Materials and Methods [file 41435_2025_356_MOESM1_ESM.docx]

**Supplementary Materials and Methods**

###

### Ethical considerations and study design

CeD patients were included at the Endoscopy Unit, Department of gastroenterology at Oslo University Hospital, Rikshospitalet in Oslo, Norway. The patients had been referred from their general practitioners for either clinical and serological suspicion of CeD or for conditions where CeD could not be ruled out (reflux disorder, upper abdominal discomfort etc). Written informed consent was obtained from all patients. All methods were performed in accordance with relevant regulations and guidelines. The diagnosis of CeD was done in accordance with the guidelines of the European Society for Study of Coeliac Disease^1^. The project has been approved and registered at the Regional Ethics Committee with accession numbers 6544 and 20521. A total of 113 participants were included in this study and classified into three different groups: controls (CTRL), treated CeD patients (TCD), and untreated CeD patients (UCD). Controls (n=40) consisted of volunteers undergoing upper endoscopy for complaints unrelated to CeD. TCD (n=39) were CeD patients on a GFD with a Marsh score ≤ 2. UCD (n=34) were CeD patients previously diagnosed or suspected of having CeD with a Marsh score of 3.

### Genotyping and quality control

DNA was isolated from blood samples and genotyped using Infinium Global Screening Array-24v1.0. Standard quality control (QC) procedures were used to remove low quality variant calls. Genotypes were imputed with the Michigan Imputation Server using the Haplotype Reference Consortium panel v1.172, as described previously^2^. SNPs with an imputation score < 0.8, a Hardy-Weinberg equilibrium p-value < 1×10^−4^, a call rate < 95%, or a minor allele frequency < 0.1 were filtered out.

### Preparation of small intestine biopsies

Biopsies were obtained by upper endoscopy and assessed by trained pathologists to determine Marsh scores following standard guidelines of histopathological assessment for CeD^1^. Extra biopsies were incubated under agitation in PBS+EDTA solution on ice for 5 minutes to obtain the epithelial lining. Cells were collected from the supernatant by pelleting at 300 g for 5 min and passed through a 70 μm cell strainer. A fraction of these cells was analysed by FACS, quantified by trypan blue exclusion medium. The rest was pelleted and resuspended in Lysis/Binding medium (AM1560). Lysed cells were frozen and stored until RNA extraction.

### FACS preparation and analysis

Single-cell suspensions were stained with the antibody panel depicted in **Supplementary Table 8**. Briefly, cells were centrifuged at 300 g for 5 min and then resuspended in 100 μL PBS supplemented with 2% FCS. Next, cells were stained for 30 min at 4℃, after which they were washed twice and resuspended in 400 μL PBS supplemented with 2% FCS. FACS data was generated using the BD LSR-II system (BD Bioscience) and analysed using FlowJo v10.

### RNA isolation of EL

Samples were processed and sequenced in two sequencing batches. Total RNA was extracted from 113 samples of the EL cell fraction using the mirVana™ miRNA Isolation Kit (AM1560). Up to 12 samples were processed simultaneously, ending with 10 batches of RNA extraction. The RNA was quantified, and integrity was confirmed by different approaches: Nanodrop spectrometry, Qubit RNA BR Assay Kit (Q10210), Qubit RNA HS Assay Kit (Q32852), Agilent RNA ScreenTape (5067-5576), and Agilent High Sensitivity RNA ScreenTape (5067-5579). Before library preparation, RNA integrity (RIN) and concentration was measured again and used to calculate a ratio of degradation ([RNA concentration after extraction]/[RNA concentration before library preparation]). Samples with a confirmed RIN > 6 and a concentration > 0.5 ng/µL were sequenced, resulting in a total of 90 samples that passed the thresholds to continue analysis.

*RNA library preparation*

RNA library preparation and sequencing was performed at GenomeScan B.V., Plesmanlaan 1d, 2333 BZ, Leiden. Sample preparation was performed according to the protocol "NEBNext Ultra II Directional RNA Library Prep Kit for Illumina" (NEB #E7760S/L). Briefly, mRNA was isolated from total RNA using oligo-dT magnetic beads. After fragmentation of the mRNA, a cDNA synthesis was performed. This was used for ligation with the sequencing adapters and PCR amplification of the resulting product. The quality and yield after sample preparation was measured with the Fragment Analyzer. The size of the resulting products was consistent with the expected size distribution (a broad peak between 300–500 bp). NovaSeq6000 was used for clustering and DNA sequencing following manufacturer's protocols using a concentration of 1.1 nM DNA. Image analysis, base calling, and QC were performed with the Illumina data analysis pipeline RTA (version 3.4.4) and Bcl2fastq (version 2.20).

### RNA-seq quantification and QC

The adapters for sequencing were trimmed from fastQ files and aligned to build human_g1k_v37 ensembleRelease 75 reference genome using Hisat (version 0.1.5)^3^ with default settings. SAMtools (version 1.2)^4^ was used to sort the aligned reads before gene quantification. Gene-level quantification was performed by HTSeq-count HTSeq (version 0.6.1p1)^5^ using --mode=union. QC metrics were calculated for the raw sequencing data using the tool FastQC (version 0.11.3)^6^. QC metrics were calculated for the aligned reads using Picard-tools (version 1.130)^7^.

The raw count matrix, containing 53,042 transcripts and 90 samples, was first filtered to remove zero-variance and non-expressed genes by selecting only genes that had at least 10 reads in 10 samples. This resulted in 20,498 genes for further analysis. Next, to remove sample outliers, we explored the first four PCs using the 1000 most variable genes, experimental variables, and sequencing QC metrics. In total, we removed eight outliers that showed at least three of the following criteria: outlier in the PC analysis, unique pair read percentages < 10%, unmapped read percentage > 10%, RNA concentration < 1.5 ng/µL, total number of reads > 3 million, sample present in batch 9 of RNA extraction, or ratio of degradation > 10 (calculated as [RNA concentration after extraction]/[RNA concentration before library preparation]). Y chromosome genes were also excluded from the dataset. The final dataset consisted of 82 samples and 20,468 genes.

### DE analysis

The DE effects of different conditions were quantified using the R package DEseq2 (version 1.34.0)^8^, including sex, age, sequencing batch, total reads, RNA integrity, and %GC content as covariates in the DE model. DE effects were calculated by comparing UCD vs CTRL, TCD vs CTRL, and UCD vs TCD. DE effects were filtered on having an absolute L2FC ≥ 1 and an adjusted p-value < 0.01. The remaining DE genes were used for interpretation and downstream analysis.

### Clustering of DE genes and sample groups

DE genes were clustered into groups as follows. The gene expression matrix was VST-normalised using DESeq2 (version 1.34.0)^8^. The matrix was then filtered to include only DE genes. Next, gene expression was centred to mean 0 and scaled. DE genes were clustered using k-means clustering (k=4) on a Euclidean distance matrix using the R package ComplexHeatmap (version 2.10.0)^9^. Samples were also clustered following a similar approach to that used for DE genes, but with k=3. The cluster number (k) was determined by comparing three different methods to obtain the optimal number of clusters that are biologically informative. These methods were obtaining gap statistic (500 permutations), average silhouette width, and total within sum-of-square when clustering from 1 to 10 groups. For this analysis, we used the R packages cluster (version 2.1.6) ^10^ and factoextra (version 1.0.6)^11^.

### Pathway enrichment analysis

Reactome pathways^12^ were used to identify the pathways or biological processes that were enriched for each set of genes. This analysis was performed using the R package clusterProfiler (version 3.14.3)^13^. P-values were adjusted using the Benjamini-Hochberg procedure to account for multiple testing.

### APOA4:KI67 ratio calculation

The gene expression matrix was VST-normalised using DESeq2 (version 1.34.0)^8^. The APOA4:MKI67 ratio was calculated by dividing the gene expression of *APOA4* by that of *MKI67* for each sample.

### Scoring of mucosal status in samples

The mucosal status score for each sample was calculated using the methodology implemented in the R package singscore (version 1.14) and the pipeline recommended by authors^14^. Briefly, singscore is a single-sample gene set scoring method that values individual samples without relying on other samples included in the dataset. It can use sets of genes that are up- and downregulated to score molecular phenotypes. First, the RNA matrix is ranked using the rankGenes() function. This rank matrix, along with the gene sets, is then passed to the simpleScore() function, which returns the output with scores and dispersions. As gene sets, the input included all possible combinations of DE genes included for each previously identified cluster. Since we know the direction of DE genes, we used clusters 1 and 2 as upregulated sets and clusters 3 and 4 as downregulated sets. Resulting scores ranged between -1 and 1. Values > 0 indicate an enrichment of the set of genes used as input, whereas a negative value corresponds to a depletion of genes. When only one cluster is used (i.e. only cluster 3), it is passed on to the upSet argument, which uses the cluster as an upregulated gene set. Additionally, the dispersion of scores was measured using the median absolute deviation of the gene set ranks, as described by the authors. Using singscores to distinguish between CeD conditions, we calculated the receiver operating characteristic (ROC) curve and area under the curve (AUC) using R package pROC (version 1.18.5).

### Bulk eQTL analysis

For bulk eQTL mapping, we tested for effects between genes and CeD-associated SNPs^15,16^ located within 250 kb of a gene centre. The RNA matrix was TMM-normalised using R package edgeR (version 3.36.0)^17^ and corrected for covariates (sex, age, sequencing batch, total reads, RNA integrity, %GC content, Marsh scores, condition, and APOA4:KI67 ratio), the first four PCs derived from gene expression, and four multidimensional scaling components derived from the genotype data. eQTLs were declared to be suggestive at FDR < 0.01. QTL mapping was performed using an eQTL pipeline that was described previously^18^.

### Deconvolution of eQTL effects in cell types

For this analysis, we employed the method Decon-QTL^19^, testing for the same effects as in the bulk eQTL mapping. Gene counts were log2 transformed and corrected for sex, age, sequencing batch, total reads, RNA integrity, %GC content, Marsh scores, condition, APOA4:KI67 ratio, the first four PCs derived from gene expression and four multidimensional scaling components derived from the genotype data. The corrected expression data was then exponentiated to maintain the original linear relationship across read counts and cell proportions. As cell counts, we used proportions of major immune and epithelial cells. Cell-type-mediated QTLs were considered suggestive at a p-value < 0.01.

### Statistical methods

Statistical analyses were performed in R (version 3.6.3)^20^, unless otherwise specified. Visualisation of results was done using the R package ggplot2 (version 3.3.0)^21^.

# References

1. Al-Toma A, Volta U, Auricchio R, et al. European Society for the Study of Coeliac Disease (ESsCD) guideline for coeliac disease and other gluten‐related disorders. United Eur Gastroenterol J 2019;7:583–613.

2. Das S, Forer L, Schönherr S, et al. Next-generation genotype imputation service and methods. Nat Genet 2016;48:1284–1287.

3. Kim D, Langmead B, Salzberg SL. HISAT: a fast spliced aligner with low memory requirements. Nat Methods 2015;12:357–360.

4. Li H, Handsaker B, Wysoker A, et al. The Sequence Alignment/Map format and SAMtools. Bioinformatics 2009;25:2078–2079.

5. Anders S, Pyl PT, Huber W. HTSeq-A Python framework to work with high-throughput sequencing data. Bioinformatics 2015;31:166–169.

6. Andrews S. FastQC a Quality Control Tool for High Throughput Sequence Data. 2010.

7. Anon. Picard Toolkit. 2019.

8. Love AM, Anders S, Huber W, et al. Package ‘ DESeq2 .’ 2017.

9. Gu Z. Complex heatmap visualization. iMeta 2022;1.

10. Mächler M, Rousseeuw P, Struyf A, et al. Cluster: Cluster Analysis Basics and Extensions. R Packag 2012;1.

11. Kassambara A, Mundt F. factoextra: Extract and Visualize the Results of Multivariate Data Analyses. 2020.

12. Fabregat A, Jupe S, Matthews L, et al. The Reactome Pathway Knowledgebase. Nucleic Acids Res 2018;46:D649–D655.

13. Yu G, Wang LG, Han Y, et al. ClusterProfiler: An R package for comparing biological themes among gene clusters. OMICS J Integr Biol 2012;16:284–287.

14. Foroutan M, Bhuva DD, Lyu R, et al. Single sample scoring of molecular phenotypes. BMC Bioinformatics 2018;19:404.

15. Ricaño-Ponce I, Zhernakova D V., Deelen P, et al. Refined mapping of autoimmune disease associated genetic variants with gene expression suggests an important role for non-coding RNAs. J Autoimmun 2016;68:62–74.

16. Ricaño-Ponce I, Gutierrez-Achury J, Costa AF, et al. Immunochip meta-analysis in European and Argentinian populations identifies two novel genetic loci associated with celiac disease. Eur J Hum Genet 2020;28:313–323.

17. Robinson MD, McCarthy DJ, Smyth GK. edgeR : a Bioconductor package for differential expression analysis of digital gene expression data. Bioinformatics 2010;26:139–140.

18. Zhernakova D V, Deelen P, Vermaat M, et al. Identification of context-dependent expression quantitative trait loci in whole blood. Nat Genet 2017;49:139–145.

19. Aguirre-Gamboa R, Klein N de, Tommaso J di, et al. Deconvolution of bulk blood eQTL effects into immune cell subpopulations. bioRxiv 2019;5:1–23.

20. R Core Team. R: A Language and Environment for Statistical Computing. 2019.

21. Wickham H. *ggplot2: Elegant Graphics for Data Analysis*. Springer-Verlag New York; 2016.
