# Supplementary material for: Transcriptomics and eQTLs reveal inflammatory heterogeneity in the duodenal lining in coeliac disease
Source: Genes Immun. 2025 Sep 9;26(5):519–30. doi: 10.1038/s41435-025-00356-0 (PMC12527925; doi:10.1038/s41435-025-00356-0)
Supplement: Supplementary file 2 — Overview Supplemental Data [file 41435_2025_356_MOESM2_ESM.pdf]

Supplementary Material

DATA

Supplementary Data. Matrix of RNA-seq raw counts of genes per sample.

Supplementary Materials and Methods. Extended description of materials and Methods.

FIGURES

Supplementary Figure 1. Spearman correlations between variables and PCs in our dataset (p-value < 0.01).

Supplementary Figure 2. Comparison of PCs in relation to CeD condition.

Supplementary Figure 3. Concordance between the DE gene results of UCD vs CTRL and UCD vs TCD.

Supplementary Figure 4. Results of the k-Means clustering of samples.

Supplementary Figure 5. Results of k-Means clustering analysis of DE genes.

Supplementary Figure 6. Enrichment analysis of inter-variation within TCD and UCD.

Supplementary Figure 7. Singscores of samples using clusters of DE genes.

Supplementary Figure 8. ROC and AUC using clusters of DE genes.

Supplementary Figure 9. Singscores of an external cohort using clusters of DE genes.

Supplementary Figure 10. ROC and AUC using clusters of DE genes in reference cohort.

TABLES

Supplementary Table 1. Sample sheet and QC details

Supplementary Table 2. List of DEGs: comparison between disease conditions

Supplementary Table 3. Enriched pathways of DEGs: comparison between disease conditions

Supplementary Table 4. List of DEGs: comparison between inflammation groups

Supplementary Table 5. Enriched pathways of DEGs: comparison between inflammation groups

Supplementary Table 6. Singscores of samples using DEGs

Supplementary Table 7. List of bulk and predicted-cell-type eQTLs

Supplementary Table 8. Cell-type counts by FACS analysis and antibody panel
